# Current practice of German anesthesiologists in airway management: Results of a national online survey

**DOI:** 10.1007/s00101-021-01025-3

**Published:** 2021-08-28

**Authors:** Nina Pirlich, Matthias Dutz, Eva Wittenmeier, Marc Kriege, Nicole Didion, Thomas Ott, Tim Piepho

**Affiliations:** 1 Department of Anaesthesiology, Medical Centre of the Johannes Gutenberg-University, Mainz, Germany; 2 Department of Anaesthesiology and Intensive Care, Brothers of Mercy Hospital, Trier, Germany

**Keywords:** Patient safety, Guidelines, Video laryngoscopy, Education, Difficult airway, Patientensicherheit, Leitlinien, Videolaryngoskopie, Ausbildung, Schwieriger Atemweg

## Abstract

**Background:**

There is a worldwide consensus among experts that guidelines and algorithms on airway management contribute to improved patient safety in anesthesia. The present study aimed to determine the current practice of airway management of German anesthesiologists and assess the safety gap, defined as the difference between observed and recommended practice, amongst these practitioners.

**Objective:**

To determine the effect of implementing the guidelines on airway management practice in Germany amongst anesthesiologists and identify potential safety gaps.

**Methods:**

A survey was conducted in September 2019 by contacting all registered members of the German Society of Anaesthesiology and Intensive Care Medicine (DGAI) via email. The participants were asked about their personal and institutional background, adherence to recommendations of the current German S1 guidelines and availability of airway devices.

**Results:**

A total of 1862 DGAI members completed the questionnaire (response rate 17%). The main outcome was that anesthesiologists mostly adhered to the guidelines, yet certain recommendations, particularly pertaining to specifics of preoxygenation and training, showed a safety gap. More than 90% of participants had a video laryngoscope and half had performed more than 25 awake intubations using a flexible endoscope; however, only 81% had a video laryngoscope with a hyperangulated blade. An estimated 16% of all intubations were performed with a video laryngoscope, and 1 in 4 participants had performed awake intubation with it. Nearly all participants had cared for patients with suspected difficult airways. Half of the participants had already faced a “cannot intubate, cannot oxygenate” (CICO) situation and one in five had to perform an emergency front of neck access (eFONA) at least once. In this case, almost two thirds used puncture-based techniques and one third scalpel-based techniques.

**Conclusion:**

Current practice of airway management showed overall adherence to the current German guidelines on airway management, yet certain areas need to be improved.

**Supplementary Information:**

The online version of this paper (10.1007/s00101-021-01025-3) includes the questionnaire.

## Abstract (de)

**Hintergrund:**

Es besteht ein weltweiter Konsens unter Experten, dass Leitlinien und Algorithmen zum Atemwegsmanagement zu einer verbesserten Patientensicherheit in der Anästhesie beitragen. Diese Studie beschreibt die aktuelle Praxis des Atemwegsmanagements deutscher Anästhesisten und identifiziert Sicherheitslücken, definiert als Differenz zwischen der durch den Antwortenden berichteten Praxis und Empfehlung.

**Zielsetzung:**

Ermittlung der Umsetzung der Leitlinie zum Atemwegsmanagement in der Praxis durch deutsche Anästhesisten und Identifizierung möglicher Safety Gaps.

**Methoden:**

Im September 2019 wurde eine Online-Umfrage durchgeführt. Alle registrierten Mitglieder der Deutschen Gesellschaft für Anästhesiologie und Intensivmedizin (DGAI) wurden per E‑Mail kontaktiert. Die Teilnehmer wurden nach ihrem persönlichen und institutionellen Hintergrund, der Einhaltung der Empfehlungen der aktuellen deutschen S1-Leitlinie und der Verfügbarkeit von Gerätschaften zur Atemwegssicherung befragt.

**Ergebnisse:**

Insgesamt 1862 DGAI-Mitglieder füllten die Fragebögen vollständig aus (Rücklaufquote: 17 %). Das Hauptergebnis war, dass sich die Anästhesisten größtenteils an die Leitlinie hielten, jedoch zeigten bestimmte Empfehlungen, insbesondere in Bezug auf die Besonderheiten der Präoxygenierung und der Ausbildung, eine Sicherheitslücke. Mehr als 90 % der Teilnehmer verfügten über ein Videolaryngoskop und die Hälfte hatte mehr als 25 Intubationen im Wachzustand mit einer flexiblen Optik durchgeführt. Allerdings verfügten nur 81 % über ein Videolaryngoskop mit stärker gekrümmtem Spatel. Schätzungsweise 16 % aller Intubationen erfolgten mit einem Videolaryngoskop, und jeder vierte Teilnehmer hatte damit eine Intubation im Wachzustand durchgeführt. Fast alle Teilnehmer hatten Patienten mit erwartet schwierigen Atemwegen betreut. Die Hälfte der Teilnehmer war bereits mit einer CICO-Situation konfrontiert und jeder fünfte musste mindestens einmal eine Notfallkoniotomie durchführen. In diesem Fall verwendeten fast zwei Drittel Punktionstechniken und ein Drittel ein Skalpell.

**Schlussfolgerung:**

Die derzeitige Praxis des Atemwegsmanagements zeigte insgesamt eine gute Übereinstimmung mit den Empfehlungen der aktuellen deutschen Leitlinie zum Atemwegsmanagement, dennoch besteht insbesondere bei der Ausbildung Verbesserungsbedarf.

## Treten Sie in den Austausch

Diese Arbeit wurde für *Der Anaesthesist* in Englisch eingereicht und angenommen. Die deutsche Zusammenfassung wurde daher etwas ausführlicher gestaltet. Wenn Sie über diese Zusammenfassung hinaus Fragen haben und mehr wissen wollen, nehmen Sie gern in Deutsch über die Korrespondenzadresse am Ende des Beitrags Kontakt auf. Die Autor*innen freuen sich auf den Austausch mit Ihnen.

## Brief introduction to the topic

Germany was one of the first countries to develop national guidelines on airway management in 2004 [5, 10]. These guidelines were compiled by an expert group based on a nationwide survey and the current evidence in the literature. The updated guidelines from 2015 integrate currently recommended techniques and strategies and represent a consensus of an expert group corresponding to level S1 of the classification levels of the Association of the Scientific Medical Societies in Germany [20]. In addition to 43 recommendations, the guidelines contain algorithms for the in-hospital management of anticipated and unanticipated airway problems and for safe extubation in adults.

## Background

There is consensus among experts that algorithm-based strategies on airway management are one of the factors contributing to improved patient safety. Nonetheless, the safety gap, defined as the difference between observed practice and ideal practice, exists, as exemplified by Cook et al. in their survey on the impact of the National Audit Program 4 (NAP4) on airway management practice in the United Kingdom [7].

Over the last decade, novel airway devices have been developed and techniques established, yet differences in equipment and concepts for airway management persist amongst German anesthesiologists. Unfortunately, audit projects such as NAP4 [8] or the Danish Anaesthesia Database [2] that examined nationwide airway management-related data and complications do not yet exist in Germany.

In order to evaluate the impact of the German airway management guidelines in terms of quality and scientific evidence, an assessment of the accordance between current airway practice and expert opinion must be made [20]. Therefore, the present survey aimed to delineate the current airway management practice in Germany.

## Methods

Ethics approval was deemed not required by the local ethics committee due to the absence of identifiable data.

### Online survey

On behalf of the scientific working group airway management (Wissenschaftlicher Arbeitskreis Atemwegsmanagement, WAK) of the German Society of Anaesthesiology and Intensive Care Medicine (DGAI), a survey with 39 questions investigating the practice of anesthesiologists in airway management was developed (see questionnaire, Electronic Supplemental Material).

To improve the quality of the survey questions, we conducted a modified cognitive pretesting. The first draft of the questionnaire was pretested by conducting semi-structured interviews with 10 physicians with different educational levels. We made corrections concerning the comprehensibility of some questions when they were uploaded to the SurveyMonkey® platform (SurveyMonkey®, San Mateo, CA, USA). After pretesting, the questions were uploaded to the SurveyMonkey® platform. An email containing the link to the platform was sent to all registered 12,164 members of the German Society of Anaesthesiology and Intensive Care Medicine in September 2019 and 4 weeks later, a reminder was sent to the members. The survey closed on 1 November. Only complete questionnaires were included in the evaluation.

### Statistical analysis

The survey results were entered into Excel 2016® (Microsoft Corporation, Redmond, USA) data sheets. Data analysis was conducted with SPSS® version 26.0 (IBM Corporation, Armonk, NY, USA). Results were presented in absolute numbers and as percentage of respondents. Data were further stratified by the training status of the participants and type of institution (hospital/private practice) and χ^2^-tests were used to compare categorical variables. A *p*-value <0.05 was considered significant. The safety gap for 15 recommendations was defined as the difference between practice and 100% compliance with a recommendation and its median [7]. It therefore indicates the no-compliance of participants with the recommendations of the guidelines. The median was calculated based on all safety gaps for the assessed recommendations for all participants (not stratified by doctor in training/experienced specialist). Vice versa, the average percentage of adherence to guidelines was calculated.

## Results

### Respondent characteristics

A total of 10,982 DGAI members received the e‑mail with the link to the survey platform, 4627 (42.1%) opened the e‑mail (checked by database administrators) and 2160 (19.7%) edited the survey within 3 months. Overall 1862 questionnaires were entirely completed and analyzed (response rate 17%). Respondent characteristics are shown in Table 1.

**Table 1:** Clinical background of the study sample (respondents = 1862)

Characteristic data	Number	(%)^a^
*Gender*		
Female	636	34.2
Male	1216	65.3
Agender	10	0.5
*Training status*		
Qualified specialist	1522	81.7
*Experience*		
<5 years	301	16.2
5–10 years	306	16.4
11–20 years	508	27.3
21–30 years	294	15.8
>30 years	113	6.1
Doctor on speciality training	340	18.3
*Professional position*		
Head of department	215	11.5
Consultant	829	44.5
Trainee	818	43.9
*Type of hospital*		
University hospital	506	27.2
Tertiary care hospital	372	20
General hospital	622	33.4
Unallocated	237	12.7
*Private practice*	125	6.7

^a^Percentages may not add up exactly to 100% due to rounding effects

### Availability of devices

**Fig. 1:**
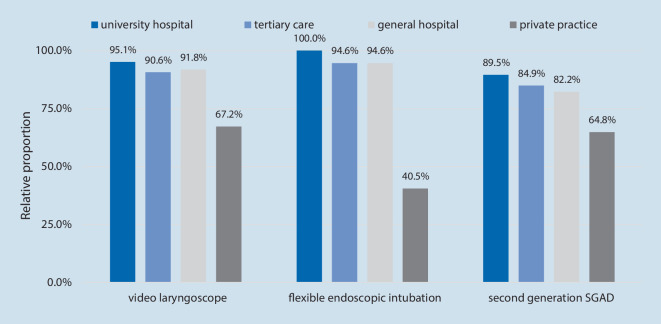
Availability of devices by type of institution

Of the survey participants 90.9 % have a video laryngoscope readily at hand at each anesthesiologist’s workstation (Fig. 1). Equipment for flexible endoscopic intubation is available for 94.1% of the survey participants, 81% of the respondents are equipped with hyperangulated blades and 83.4% of the survey participants stated that second generation laryngeal masks (SGAD) are available at their workstation.

### Strategies of airway management

**Fig. 2:**
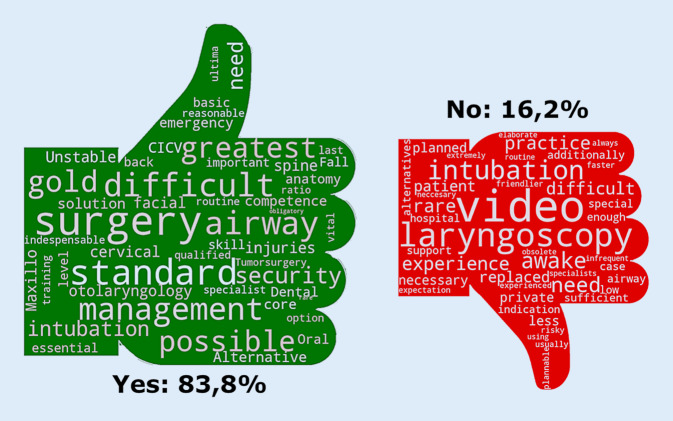
The reasons for (*Yes*) and against (*No*) awake tracheal intubation (*ATI*) visualized in word clouds. Proportion of respondents (%)

The estimated proportion of video laryngoscopy of the total number of intubations of all survey participants is 16.2%. Approximately one quarter (25.4%) of the respondents performed awake video laryngoscopies and 83.8% of the respondents regarded the experience to perform an awake tracheal intubation (ATI) as important. The reasons for and against ATI are visualized in word clouds (Fig. 2).

Of all respondents 60.8% felt that they have sufficient experience to perform ATI (69.5% of the qualified specialists and 22.1% of the doctors on specialty training). Approximately half of the survey participants (51.1%) have performed more than 25 ATI, of which 60.7% are qualified specialists.

### Management of the difficult airway

**Fig. 3:**
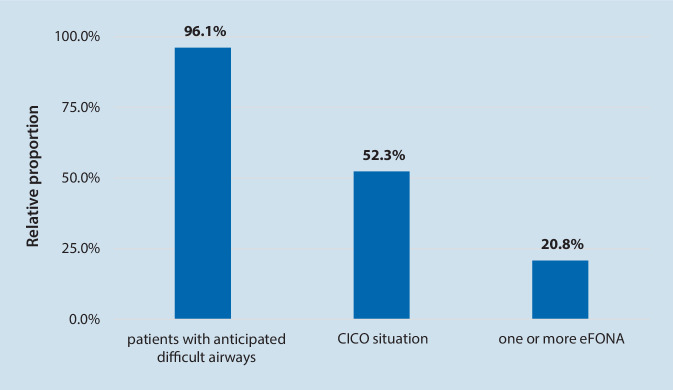
Experience of participants with difficult airways (CICO = cannot intubate, cannot oxygenate; eFONA = emergency front of neck access)

Almost all survey participants treated patients with anticipated difficult airways (Fig. 3). More than half of the survey participants had to deal at least with one cannot intubate, cannot oxygenate (CICO) situation in daily practice. Nearly one fifth of the participants had to perform one (14.8% of the participants) or more (6%) emergency front of neck access (eFONA) in their working life, 59.6% used puncture-based techniques (catheter over needle or Seldinger technique) and followed by the scalpel-based technique (40.3%).

### Safety gap

**Fig. 4:**
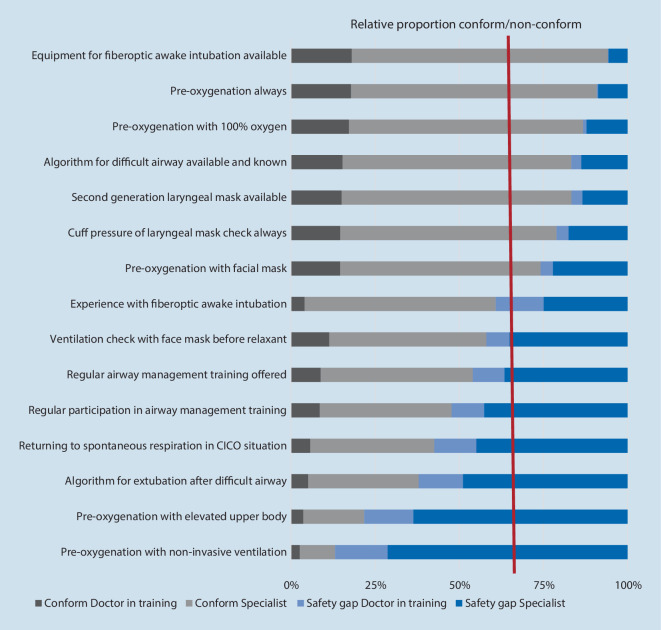
Safety gaps identified for recommendations of the German guidelines. *Red line* indicates the median safety gap (39.2%)

Of the survey participants 90.9% were aware of the S1 guidelines on airway management of the DGAI. The safety gaps identified for the 15 assessed recommendations of the guidelines are presented in Fig. 4. Qualitative responses were not represented in the figure and may be obtained from the corresponding author upon request. The greatest safety gap was identified for the pre-oxygenation with noninvasive ventilation (86.9%), followed by pre-oxygenation with an elevated upper body (78.2%) and availability of an algorithm for extubation after a difficult airway (62.0%). The smallest safety gap was observed for availability of equipment for fiberoptic awake intubation, with only 5.9%. Practitioners in training responded significantly more frequently with “yes” to the questions on whether a preoxygenation is always performed (*p* < 0.001), preoxygenation is performed with 100% oxygen (*p* < 0.001), and whether a tightly fitting mask is used for preoxygenation (*p* < 0.01).

## Discussion

The current practice of airway management amongst German anesthesiologists following implementation of national guidelines has not previously been described. The main outcome of the survey was that German anesthesiologists mostly adhered to the existing guidelines, yet certain areas showed a safety gap and indicated the need for improvement. It was observed that German anesthesiologists are well-equipped with video laryngoscopes and equipment for flexible intubation but lack education of relevant concepts and training of specific techniques.

The first German survey focussed on the availability of specialized equipment for difficult airway management in obstetric units [22]. A difficult airway cart was available in almost all of the departments. The first comprehensive data on airway management in Germany were collected in 2006 from directors of university hospitals or affiliated hospitals, before implementing the first version of the German guidelines and hence providing a basis for the guidelines [13]. In contrast, the respondents of the present survey were 2160 individual anesthesiologists. Results of an online survey conducted in 2010 that assessed the procurement, storage and preference of airway management devices by anesthesia departments in German hospitals also confirmed that German hospitals are well-equipped with airway management devices [24].

Schiff et al. conducted the first study assessing incidents and complications in otherwise healthy patients undergoing elective procedures based on 1.37 million anesthetic procedures from a national outcome-tracking database [21]. Patients with an American Society of Anesthesiologists (ASA) class I and II treated in Germany between 1999 and 2010 were analyzed. The calculated rate of anesthesia-related death due to airway management problems was 15%. In the present survey, more than half of the survey participants (52.3%) had to deal at least with one CICO situation in their professional career. This is equivalent to data from a Canadian national survey of the year 2005, in which 57% of the respondents encountered a CICO [25].

Adherence and implementation data on airway management guidelines remain limited [10]. Considering the importance of compliance with the guidelines for patient outcomes, we assessed the accordance between current airway practice and recommendations. While over 90% of the survey participants were aware of the current guidelines, the adherence to certain recommendations was below one quarter. It can be speculated that the reasons for this are missing national audit projects or registers and insufficient training.

The availability of a video laryngoscope was determined for 90.9% of anesthesiologists responding in the present survey. This demonstrates good adherence to the recommendation that a video laryngoscope must be at hand and readily available. In comparison to data from other European countries, the adherence of German anesthesiologists to the guidelines is astoundingly high. In Sweden, only half of the anesthesiologists (56.8%) reported having immediate access to video laryngoscopes in the year 2018 [4]. Gill et al. assessed the equipment of anesthetists in the United Kingdom as part of two surveys conducted in 2010 and 2015 and discovered that only 57.5% of respondents, who were members of the Difficult Airway Society (DAS), had a video laryngoscope [12]. Cook et al. determined in their 2014 survey the availability of video laryngoscopes in a sample of all United Kingdom National Health Service Hospitals and reported a considerably higher rate of availability of 91%, which is comparable to the proportion determined amongst German anesthetists [6].

A survey conducted amongst German anesthesiologists determined that a fiberoptic bronchoscope was available in 85.9% of obstetric units [22]. At the same time, some anesthesiologists advocate the replacement of the bronchoscope by modern techniques [1]. The present survey shows that German anesthesiologists emphasize awake flexible intubation endoscopy in cases of a known difficult airway. Anesthesiologists today are presumably not trained in this technique, as it is increasingly being replaced by video laryngoscopy: every fourth participant used a video laryngoscope for ATI. In the present study, 85.9% stated to consider ATI the gold standard for the expected difficult airway, and 94.1% had a bronchoscope at hand. It is unclear how the remaining 15% of anesthesiologists handle a difficult airway situation, but it is possible that they may not conduct an elective anesthesia in such situations.

The Helsinki declaration on patient safety in anesthesiology has clearly opted for guidelines on airway management [14]. The algorithm must be guided by local circumstances and existing preferences and undergo regular adaptations and updates. This is of particular importance for the safety of these patients, as it was demonstrated that the implementation of guidelines and knowledge thereof could lower the incidence of adverse events due to airway management [9]. Therefore, the present study adds to the assessment of compliance with current guidelines by German anesthesiologists. It was observed that while compliance with several recommendations of the guidelines was high (particularly those pertaining to equipment for awake intubation, pre-oxygenation and knowledge of difficult airway management), noncompliance with certain recommendations of the guidelines was identified, with a median safety gap of 39.2% (Fig. 4). This median is almost identical with the median safety gap of 40% reported by Cook et al. for the NAP4 guidelines [7]. In contrast, the type of the safety gaps differed between both surveys. The largest safety gaps observed in the present study were regular participation in airway management training, pre-oxygenation with noninvasive ventilation, pre-oxygenation with an elevated upper body, and an algorithm for extubation after difficult airway management (Fig. 4), while Cook et al. reported the largest safety gaps for CICO training and cricothyroidotomy training and available policy for failed intubation. Interestingly, in both studies noncompliance with the recommendations of the respective guidelines was linked to training of particular techniques, indicating a need for more specific and more frequent training opportunities of such techniques.

It is surprising that only 58% of the survey participants follow the recommendation proving feasibility of mask ventilation prior to neuromuscular block is not necessary [18]. Also, 65.1% Swedish anesthetists used a mask ventilation trial [19]. A recent well-performed investigation confirmed the results of many further studies, showing that the use of a neuromuscular block improves mask ventilation [15]. The survey data demonstrated the presence of myths concerning airway management and the long delay between publication and its adoption into clinical practice. The comparison of the surveyed CICO rate of 52.3% with the cricothyrotomy rate of 20.8% of this survey might explain the opinion of 42.6% of the respondents, which suspect the return to spontaneous respiration in a CICO situation as realistic. Interestingly, this recommendation is also given by guidelines of other countries [19] despite the myth of returning to spontaneous respiration in a CICO situation was scientifically refuted in several studies [15]. Pharmacologic interventions cannot be relied upon to rescue patients in a CICO scenario. In our opinion, it should not be recommended in guidelines because of its passive character.

In the study by Ott et al., 11 of 40 participants had already performed an eFONA in real patient care. The authors were surprised about the high rate (27.5%) and underlined as cause a potential bias of their sample, as experienced providers could have more quickly decided to perform a cricothyrotomy [16]. The current rate of this survey was approximately one fifth. There is no recommendation of the optimal technique for cricothyrotomy given in the S1 guidelines [17]. In contrast, in the DAS guidelines for the management of unanticipated difficult intubation in adults, a scalpel-bougie technique is recommended [11]. The authors decided to determine a first-line technique with the intention to provide safety by standardization in a rare crisis situation. Anesthesiologists of different countries have demonstrated a preference in an editorial advocating a single technique, recommending the scalpel-based technique [3]. In contrast, the use of a scalpel is not explicitly recommended in the German S1 guidelines. It is argued that the dependence of scalpel use on familiarity with the technique and appropriate decision-making may render the puncture-based technique more practicable [23].

Nearly one fifth of the participants had to perform one or more eFONA in their working lives. Puncture-based techniques are the most used technique, followed by scalpel-based technique. The NAP4, a registry of the major complications of airway management, recorded details of serious adverse events over a 12-month period in England. Of these, 58 involved an attempt at a cricothyrotomy or urgent tracheostomy. Therefore, the frequent use of puncture-based techniques by German anesthesiologists is in contrast to NAP4-based recommendations in the guidelines. Discussion in the literature has dealt with the optimal equipment, training and human factors [11].

Another recommendation of the guidelines is the implementation of education and regular training to ensure the successful management of unexpected and expected difficult airways [17]. Nonetheless, less than half of the participants stated that they regularly take part in airway workshops and in-hospital training workshops are offered for slightly more than half of the respondents. Although this work concentrates on the German experience, the lack of education and training seems to be a global problem [10, 20, 21].

The German situation was already described in 2017 with dissatisfaction among doctors on speciality training as well as criticism of the system concerning training conditions. It is noticeable that only half of the anesthesiologist, with almost two thirds being specialists, stated to have performed more than 25 ATI, yet this is a requirement for completion of anesthesia specialist training. It is apparent that ATI should be trained more, e.g., in simulations [26]. The demographic change and progressing economization in German hospitals lead to this decline in education and training. In addition, German anesthesiologists must cover external training costs, such as workshop participation, mostly themselves. Efforts of quality management, such as certificate workshops, should ensure practice quality and a structured guideline-appropriate approach.

### Limitations

The present survey has some limitations that should be addressed. First, the data are based solely on questionnaires and hence reflect personal, subjective opinions. Some phrasing, i.e., product names or terminology, was not explicitly defined and thus open to the respondent’s interpretation. As with all online surveys, anonymity does not guarantee the correctness of the given information and repeated survey participation cannot be ruled out. In addition, not all recommendations and aspects of current airway management could be included in the survey. For example, most participants were specialists and trainees were a minority. Therefore, relevant experiences of the trainees were not assessed in detail, e.g., experience in ATI. There are no other available data on current practice of German anesthesiologists in airway management.

## Conclusion

- This survey was conducted to highlight topics of current airway management of potential improvement and to give an impulse for adopting new technologies and techniques with the outdated ones.
- Our findings suggest that there was overall a moderate adherence to the current guidelines in airway management, yet certain areas need to be improved.
- The influence of personal experience, institutional requirements and available equipment and skills corroborate the importance of training and education with respect to the existence of algorithms and guidelines.
- The advantage of assessing the current situation of anesthesiology practice in Germany and identifying safety gaps pertaining to particular guidelines is the chance to increase awareness for areas that need to be improved and reconsidered in future guidelines.

## Supplementary Information

Questionnaire
