# Supplementary material for: Current practice of German anesthesiologists in airway management: Results of a national online survey
Source: Anaesthesiologie. 2021 Aug 28;71(Suppl 2):190–7. doi: 10.1007/s00101-021-01025-3 (PMC9763150; doi:10.1007/s00101-021-01025-3)
Supplement: Supplementary file 1 — Questionnaire [file 101_2021_1025_MOESM1_ESM.pdf]

Questionnaire to " Current practice of German anaesthesiologists in airway management. Results of a national online survey " by Pirlich N, Dutz M, Wittenmeier E et al. (2021) in *Der Anaesthesist*.
Article and supplementary material are available at www.springermedizin.de. Please enter the title of the article in the search field

Questionnaire

1. Please indicate your gender:

- ☐ female
- ☐ male
- ☐ agender

2. You are ...

- ☐ doctor in specialty training
- ☐ experienced specialist

3. If you have chosen specialist in question 2: How many years of experience as a specialist do you have?

- ☐ < 5 years
- ☐ 5-10 years
- ☐ 11-20 years
- ☐ 21-30 years
- ☐ > 30 years

4. Do you have leading position?

- ☐ No
- ☐ Yes, as a senior physician (“Oberarzt”)
- ☐ Yes, as a senior consultant (“Chefarzt”)

5. Where do you work?

- ☐ university hospital
- ☐ tertiary care hospital
- ☐ general hospital
- ☐ teaching hospital
- ☐ private practice

6. How many work areas for anaesthesiology are in your hospital/practice?

☐ <5
☐ 5-10
☐ 11-20
☐ >20

7. In your workplace, is general anaesthesia regularly required for

☐ surgeries performed by otolaryngologists
☐ surgeries performed by oral and maxillofacial surgeons
☐ surgeries performed by dentists
☐ bariatric surgery
☐ pulmonary patients
☐ none of the above

8. In the past, did you experience an in-hospital 'cannot-intubate, cannot-oxygenate'-situation?

☐ never
☐ once
☐ more than once
☐ I do not know

9. Do you care for patients with known difficult airway?

☐ yes
☐ no

10. Which screenings do you perform at a preoperative visit? (multiple answers allowed)

☐ Mallampati test
☐ thyreomental distance
☐ upper lip bite test
☐ neck mobility test
☐ palm print test
☐ mouth opening
☐ Wilson risk score
☐ Arne risk index

☐ El-Ganzouri risk index
☐ other (please specify)

11. Before inducing a general anaesthesia, do you perform a pre-oxygenation on a spontaneously breathing patient?

☐ yes, always
☐ only for risk patients
☐ only if the patient can tolerate it
☐ only if the time allows
☐ never

12. I pre-oxygenate the patient…. (multiple answers allowed)

☐ with an elevated upper body
☐ with a closely fitted face mask
☐ with 100% of oxygen
☐ with < 100 % oxygen, to avoid resorption atelectases
☐ with 8 deep breaths within 60 seconds
☐ until the expiratory measured oxygen concentration is above 90%
☐ by applying non-invasive ventilation (e.g. pressure support: 8 cmH_2O, PEEP 5 cmH_2O)

13. Before applying the muscle relaxant, do you test the ventilation with a face mask?

☐ yes
☐ no

14. Supraglottic Airway Devices (SGAD)

a) To which SAD do you have access at your in-hospital setting?

☐ First generation laryngeal mask
☐ Second generation laryngeal mask (with gastricdrainage channel)
☐ First generation laryngeal tube
☐ Second generation laryngeal tube (with gastric drainage channel)
☐ intubation laryngeal mask iLMA
☐ other intubation laryngeal mask
☐ combitube

b) Do you check the cuff pressure of the SGAD?

☐ yes, always
☐ occasionally, if a cuff pressure device is available
☐ no

c) **Do you perform regular tests to check the correct position of your SGAD?**

☐ correct insertion depth
☐ sufficient ventilation
☐ "bubble test"
☐ supra-sternal notch test
☐ non-resistant insertion of a stomach tube and suction of gastric content

d) **Do you use the second generation SGAD regularly for the following extended indications:**

☐ duration of SGAD > 2 hours
☐ laparoscopic surgery
☐ BMI > 30 kg/m^2
☐ prone position
☐ adenotomy or tonsillectomy of children
☐ gastroesophageal reflux (food-dependent)
☐ gastroesophageal reflux (daily)
☐ none of the above

e) **In your clinical practice, do you train the intubation via laryngeal mask on patients with inconspicuous airway?**

☐ yes
☐ no

f) **Do you use the laryngeal tube in your clinical practice?**

☐ no
☐ yes, as an alternative to LMA
☐ only for training purposes

15. Which blade shapes are available at your anaesthesiologist work station to perform a direct laryngoscopy?

☐ straight blade (e.g. Miller)
☐ curved blade (e.g. Macintosh)
☐ McCoy blade (with a mobile tip to elevate the epiglottis)
☐ others: ____

16. Video laryngoscopy (VL)

a) **Please estimate the proportion of video laryngoscopies of all intubations in your hospital?**

☐ 100 % ☐ 90 % ☐ 80 % ☐ 70 % ☐ 60 %
☐ 50 % ☐ 40 % ☐ 30 % ☐ 20 % ☐ 10 % ☐ 0 %

b) **Do you have a video laryngoscope readily at hand at each of your anesthesiologist work stations?** **(readily = in time according to your opinion)**

- ☐ yes
- ☐ no

c) **Which video laryngoscope do you have at your working area?**

- ☐ none
- ☐ video laryngoscope with Macintosh (or similar) blade
- ☐ video laryngoscope with straight blade (Miller)
- ☐ video laryngoscope with highly curved blade
- ☐ others (please specify)

d) **Do you perform awake video laryngoscopies?**

- ☐ yes
- ☐ no

e) **Do you consider the establishment of defined criteria to perform a video laryngoscopy (i.e., a standard operating procedure) valuable?**

- ☐ yes
- ☐ no

17. **Training**

a) **Do you regularly participate in airway management training?**

- ☐ yes
- ☐ no

b) **Does your clinic provide a regular airway management training/simulation (information on the available equipment)?**

- ☐ yes
- ☐ no

18. **Do you have rigid endoscopes (e.g. Bonfils)?**

- ☐ yes
- ☐ no

19. **Awake fiberoptic intubation**

a) **Do you have the equipment for the fiberoptic awake intubation in your hospital or private practice?**

- ☐ yes
- ☐ no

b) Do you think that your experience with awake fiberoptic intubation suffices to feel comfortable with this skill?

☐ yes

☐ no

c) How many awake fiberoptic intubations have you performed yet?

☐ 0 ☐ < 10 ☐ 10-25

☐ 26-50 ☐ > 50

d) Do you think that the expertise to perform an awake intubation is important for your daily routine? Please choose yes or no and explain your choice.

☐ yes, because….

☐ no, because….

20. Emergency Front of Neck Access (eFONA)

a) How often have you performed an in-hospital eFONA?

☐ never

☐ once

☐ several times

b) Which technique of eFONA do you prefer in your clinical setting under consideration of your available equipment?

☐ scalpel technique (scalpel, speculum, tube)

☐ cannula based (e.g. Quicktrach)

☐ Seldinger technique (e.g. Melker Set)

☐ others: ___________________________________

21. Do you have an algorithm of difficult airway and is it known by and accessible to all members of your hospital?

☐ yes

☐ no

22. Do you believe that a return to spontaneous breathing is possible in case of a 'cannot-intubate/cannot-oxygenate' situation?

☐ yes

☐ no

23. **<u>Extubation:</u> Do you have an algorithm for a planned extubation after difficult airway management?**

☐ no

☐ yes: ____

24. **Do you know a guideline on airway management?**

☐ no

☐ S1 Guidelines of the German Society of Anaesthesiology and Intensive Care Medicine (DGAI)

☐ Difficult Airway Society (DAS) Guidelines

☐ others: _______________________________
